# A feasibility study on non-invasive oxidative metabolism detection and acoustic assessment of human vocal cords by using optical technique

**DOI:** 10.1038/s41598-017-16807-2

**Published:** 2017-12-05

**Authors:** Tzu-Chieh Lin, Jung-Chih Chen, Chih-Hsien Liu, Chia-Yen Lee, Yung-An Tsou, Ching-Cheng Chuang

**Affiliations:** 1 Institute of Biomedical Engineering, National Chiao Tung University, Hsinchu, 30010 Taiwan; 2 Department of Otolaryngology, China Medical University Hospital, Taichung, 40447 Taiwan; 3 Department of Otolaryngology, Hsinchu Cathay General Hospital, Hsinchu, 30060 Taiwan; 4 Department of Electrical Engineering, National United University, Miaoli, 36063 Taiwan

## Abstract

Voice disorder such as vocal fatigue is a common and complex multifaceted clinical problem that presents a significant impact on quality of life. In this study, the functional near-infrared diffuse optical technique (fNIRS-DOT) was proposed as a novel approach for human vocal cords oxidative metabolism detection and acoustic assessment simultaneously to provide a multidimensional assessment of voice disorder. A totally of 60 healthy subjects included 30 male and 30 female adults of age-matched were recruited and performed a vocal loading task to trigger a mild inflammation of the vocal cords in this study. In the results of oxidative metabolism, the vocal cords expressed hypoxia after vocal loading task in both male and female groups that could provide relevant information on the relationship between tissue oxygen consumption and supply for vocal cords diagnosis. Additionally, the results of optical acoustic assessment revealed the worse/changes voice quality after vocal loading task. Therefore, integration of non-invasive oxidative metabolism detection and acoustic assessment by using optical technique could provide more relevant information for diagnosis of voice disorders. The multi-functional vocal cords detection system could provide a good feasibility for clinical applications such as diagnosis and therapeutic monitoring of voice disorder.

## Introduction

Voice disorder such as the vocal fatigue is a complex clinical phenomenon that is recognized as potentially debilitating presents a significant challenge to clinical practice^1,2^. Professional voice users such as teachers, singers and actors are particularly susceptible to vocal fatigue^3–6^. Certainly, vocal fatigue can seriously affect the social and occupational functioning of such professions. Although vocal fatigue has been defined as a feeling of vocal tiredness and weak voice after the voice is overused or abused^7–9^. Unfortunately, the definition, critical identifying features, and fundamental mechanisms of vocal fatigue remain either unclear. Therefore, vocal fatigue is an important vocal health issue that needs to be further investigated. Numerous studies have adopted the vocal loading test to induce vocal fatigue in voice-healthy participants, and then to track the changes in vocal function that has potential worth to investigate the progression of fatigue as it affects the normal voice^10–17^. The several current clinical detection methods such as auditory perceptual analysis, vocal cords imaging (laryngoscope and videolaryngostroboscopy), aerodynamic analysis, acoustic analysis, and self-evaluation have been widely used for clinical applications of the assessment of voice problems^18–23^. These methods have greatly increased our knowledge about the mechanisms of vocal fatigue. However, vocal fatigue is most likely multifaceted physiological and biomechanical mechanisms. The mechanisms include neuromuscular fatigue, increased vocal fold viscosity, reduced blood circulation, non-muscular tissue strain, and respiratory muscle fatigue. Therefore, the more multidimensional assessment of vocal fatigue is very important for clinical applications.

Functional near-infrared spectroscopy (fNIRS) is a non-invasive technique that provides continuous recording of local changes in tissue oxygenation and perfusion with detection of oxy- and deoxy-hemoglobin concentration changes with at least dual-wavelength (around 800 nm) near-infrared illumination^24,25^. Additionally, fNIRS has several benefits such as less expensive, non-ionizing radiation, real-time measurement, long-time monitoring, easy operation, and completely patient-oriented measurement with high temporal resolution (∼ms). The photons in the spectral window of 600–1000 nm wavelength (also known as “optical window” or “therapeutic window”) can penetrate several centimeters into human tissue. Therefore, fNIRS has generated a lot of scientific interest and has been applied in various deep tissue applications such as imaging of the brain, breast, limb, muscle, and joint^24–31^. According to the type of source modulation, fNIRS can be classified into three modes as continuous wave (CW), frequency domain (FD), and time domain (TD)^25,31^. In the CW system, the constant illumination is used for intensity measurement. For this reason, the CW system is solely used to detect changes in absorption coefficient that cannot be used to determine the absolute value of the concentration of oxygenated and deoxygenated hemoglobin. Contrarily, the information of absorption and scattering coefficients of the medium can be determined by using FD and TD system to measure the intensity and phase or time delay of the received light. Therefore, the absolute value of the tissue optical parameters can be estimated to obtain the absolute value of the concentration of oxygenated and deoxygenated hemoglobin. However, in the clinical studies, the analysis of statistically significant difference before and after some specific test is more important than quantification of absolute value. The CW system can be used to detect the concentration changes of oxygenated (Δ[HbO_2_]) and deoxygenated hemoglobin (Δ[Hb]). Additionally, the CW system provides higher time resolution, relatively low-cost, and easy transportability. Therefore, the CW based system was developed for detection of vocal cords in this study. Besides, the optical detection method such as fiber-optic acoustic sensing is able to measure vibrations from the surface of the skin during vocalization, providing intensity of time series and frequency information that can be processed for acoustic analysis. The fiber-optic acoustic sensing also called optical microphone has many benefits such as high sensitivity, anti-RFI, anti-EMI, high safety and credibility for medical diagnosis^32–35^. Consequently, the optical detection methods can provide not only oxidative metabolism detection but also acoustic assessment for vocal cords diagnosis.

In this study, the multi-functional vocal cords detection system was developed by using the functional near-infrared diffuse optical technique. The techniques of fNIRS and optical microphone were integrated into a novel system that provided the information of oxidative metabolism detection and acoustic assessment simultaneously. A totally 60-min vocal loading task was performed to induce vocal fatigue. Therefore, the oxygenic and acoustic information before and after vocal loading task were obtained and analyzed for investigating of vocal fatigue. The results indicated that the mild inflammation after vocal loading task induced vocal cords hypoxia. Additionally, the results of optical acoustic assessment revealed the worse voice quality after vocal loading task. Our results implied that the integration of oxygen consumption detection and acoustic assessment by using fNIRS diffuse optical technique could provide more multidimensional information for clinical applications of voice disorder.

## Results

**Figure 1:**
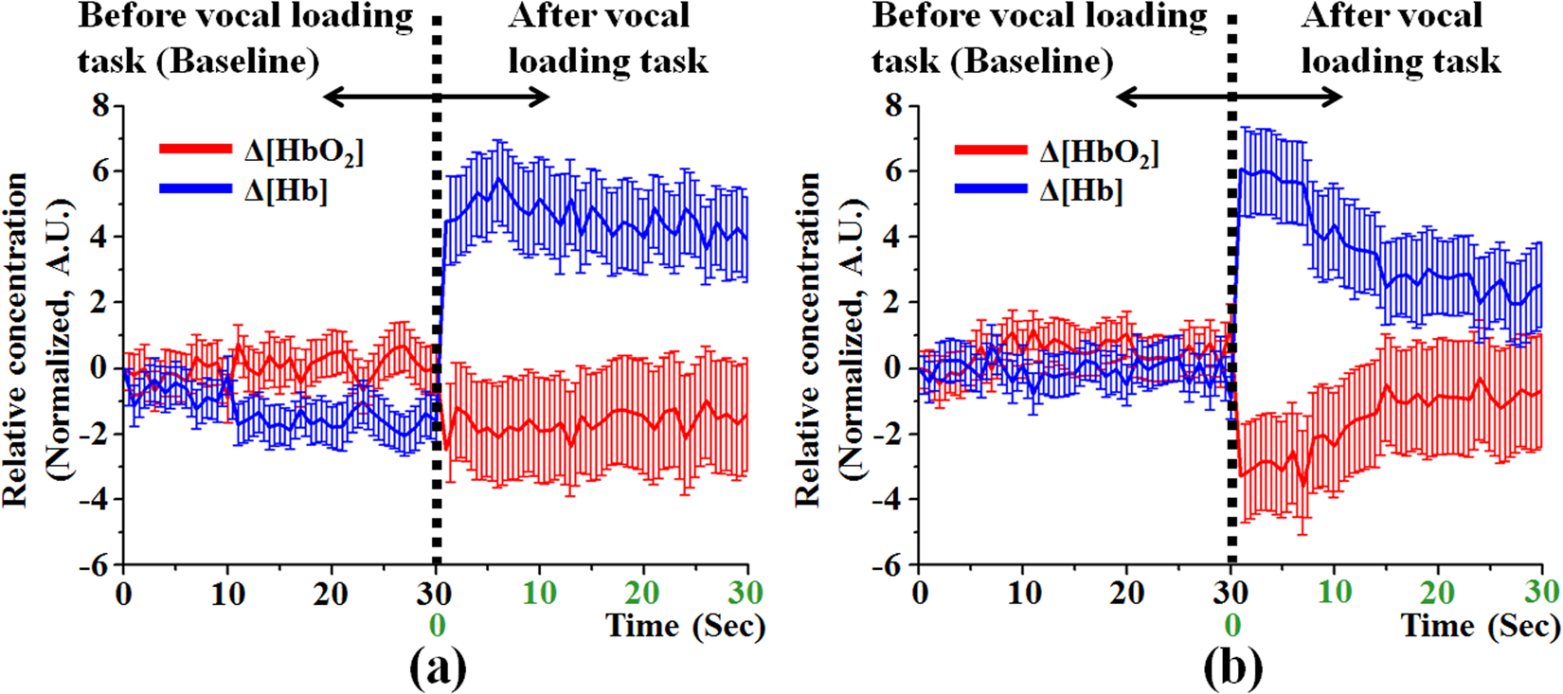
Temporal tracings of the concentration changes Δ[HbO_2_] and Δ[Hb] before and after vocal loading task. (**a**) male group and (**b**) female group.

Figure 1 shows the mean values of temporal tracings of the concentration changes Δ[HbO_2_] and Δ[Hb] before and after vocal loading task. Obviously, the temporal tracings of Δ[HbO_2_] and Δ[Hb] were significantly different between male and female groups, especially after vocal loading task. In both groups, the Δ[HbO_2_] was lower after vocal loading task than before vocal loading task. Contrarily, the Δ[Hb] was higher after vocal loading task than before vocal loading task. Additionally, the Δ[HbO_2_] was higher than the Δ[Hb] before vocal loading task and the Δ[HbO_2_] was lower than the Δ[Hb] after vocal loading task. The results indicated that the mild inflammation may induce vocal cord hypoxia. Besides, it is noteworthy that the Δ[HbO_2_] and Δ[Hb] maintained a stable trend after vocal loading task in male group (as shown in Fig. 1(a)). In the female group, the Δ[HbO_2_] slowly increased and the Δ[Hb] slowly decreased to a stable state after vocal loading task (as shown in Fig. 1(b)). Compared with female group, the male group presented higher Δ[Hb] after vocal loading task. The results suggested that the vocal cord hypoxia after vocal loading task was stronger in male group than in the female group.

**Figure 2:**
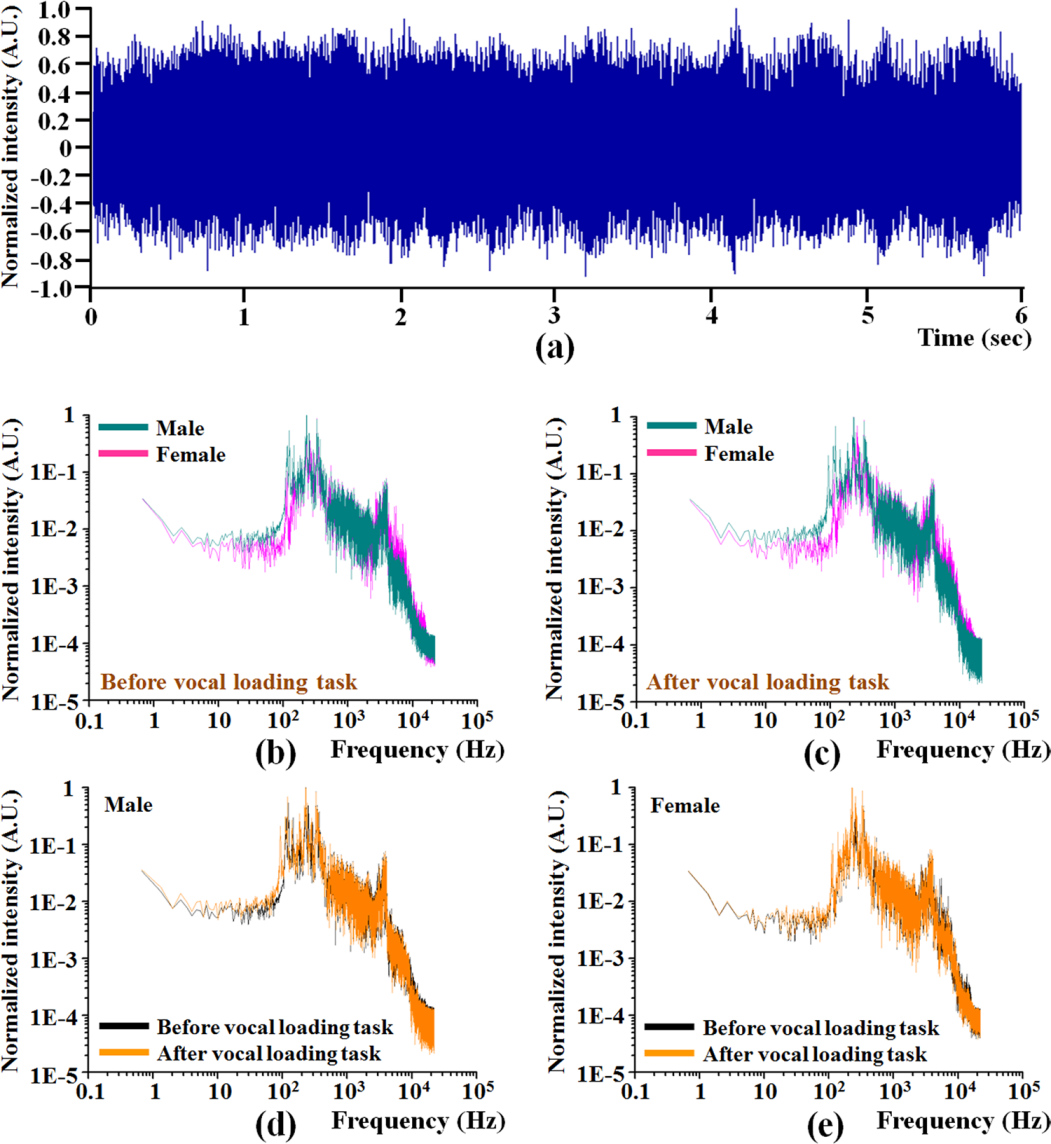
The distribution of time series optical signals and the frequency spectrum with logarithmic scale. (**a**) the time series optical signals of the tissue surface vibration during subjects voiced the sustained vowel /â/; (**b**) the frequency response in the step before vocal loading task of male and female groups; (**c**) the frequency response in the step after vocal loading task of male and female groups; (**d**) comparison of frequency response before and after vocal loading task in male group; (**e**) comparison of frequency response before and after vocal loading task in female group.

In this study, the time series optical signals of the tissue surface vibration during subjects voiced the sustained vowel /a/ were detected for acoustic analysis (as shown in Fig. 2(a)). Figure 2(b) to (e) show the frequency spectrum with a logarithmic scale that was processed from the time series optical signals by using the algorithm of fast Fourier transform (FFT). Figure 2(b) and (c) shows the comparison of frequency spectrum between male and female groups before and after vocal loading task. Although the male and female groups showed a rather similar distribution of the frequency spectrum, the response spectrum was significantly different in regions with the frequency below 1 kHz. Figure 2(d) shows the comparison of frequency spectrum before and after vocal loading task in male group. Similarly, Fig. 2(e) shows the comparison of frequency spectrum before and after vocal loading task in female group. Obviously, the vocal loading task caused the variation in regions with low frequency. The results suggested that the vocal loading task could indeed trigger a mild inflammation of the vocal cords and make the voice hoarse and deeper.

**Figure 3:**
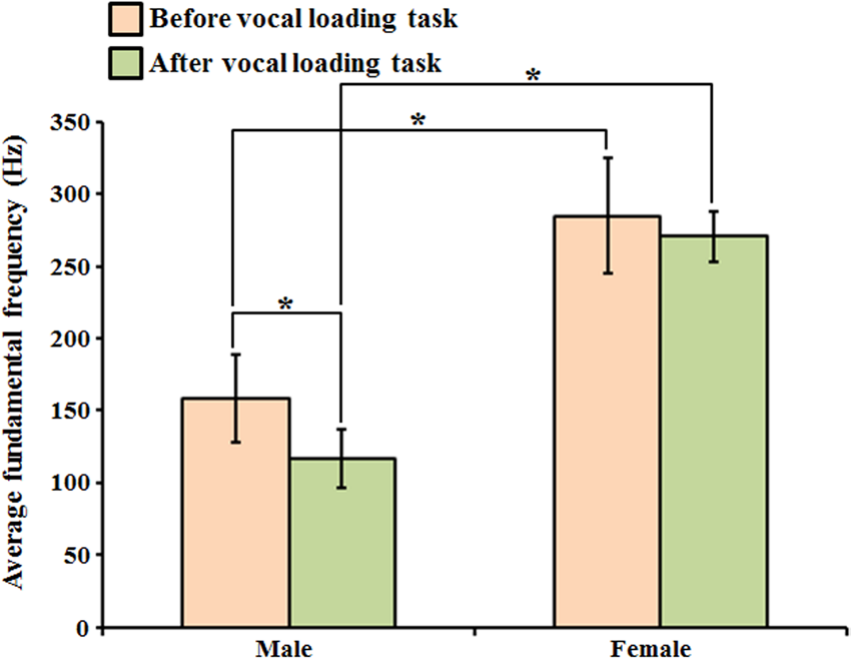
The analysis of average fundamental frequency variation before and after vocal loading task in male and female groups. (two sample *t*-test *p < 0.01).

Figure 3 shows the variation of average fundamental frequency before and after vocal loading task in the male and female groups. The average fundamental frequency in male group was lower than in female group. Additionally, the result indicated that the average fundamental frequency was significantly different between the male and female groups whether before or after vocal loading task. In the male group, the result of average fundamental frequency was significantly different before and after vocal loading task. However, the average fundamental frequency was not significantly different before and after vocal loading task in female group. Nevertheless, the fundamental frequency was lower after vocal loading task in both male and female groups. The result implied the worse voice quality after vocal loading task.

**Figure 4:**
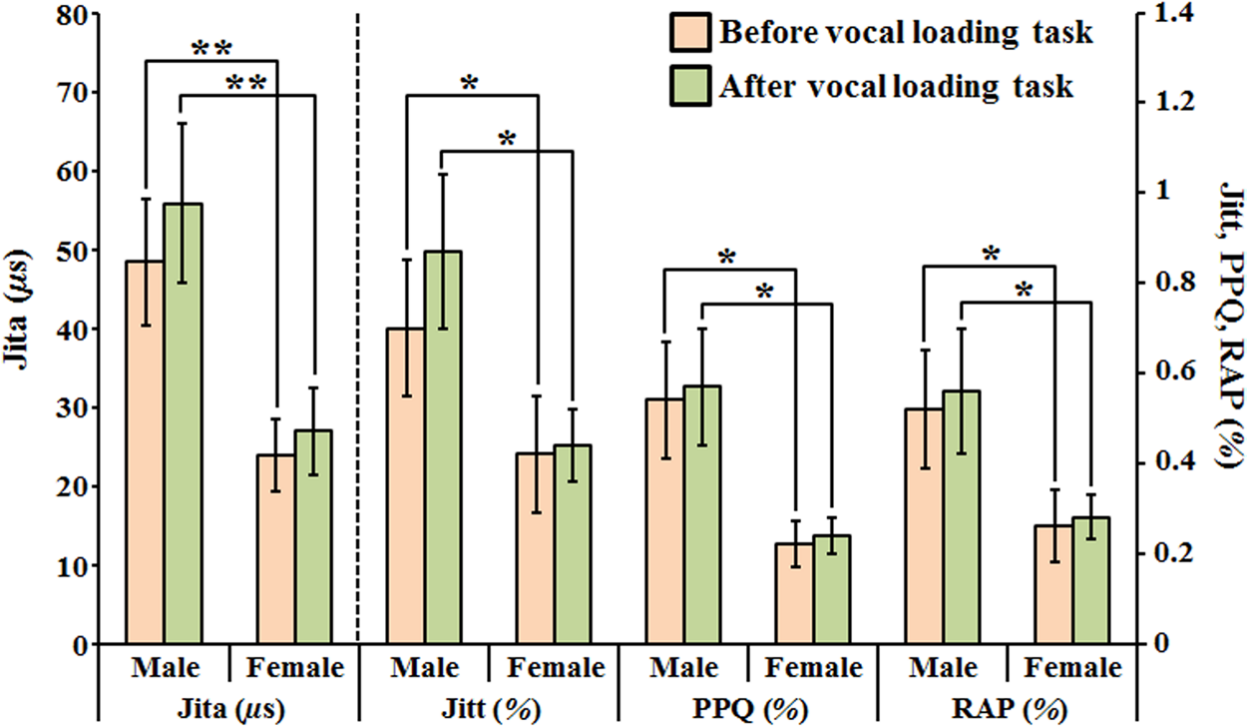
The analysis of the variation of parameters related to frequency perturbation before and after vocal loading task in male and female groups. (two sample *t*-test *p < 0.01, **p < 0.001).

In this study, the acoustic parameters related to frequency perturbation were calculated to evaluate the differences between male and female groups. Additionally, the differences before and after vocal loading task were also analyzed in male and female groups, respectively. Figure 4 shows the variation of the parameters related to frequency perturbation. The results indicated that all the values of parameters in the male group were higher than in the female group whether before or after vocal loading task. Obviously, all the parameters were significantly different between the male and female groups. However, the result presented no significant difference before and after vocal loading task in the male group or in the female group. It is still noteworthy that all the value of parameters increased after vocal loading task. The increase in frequency perturbation represents the worse voice quality.

**Figure 5:**
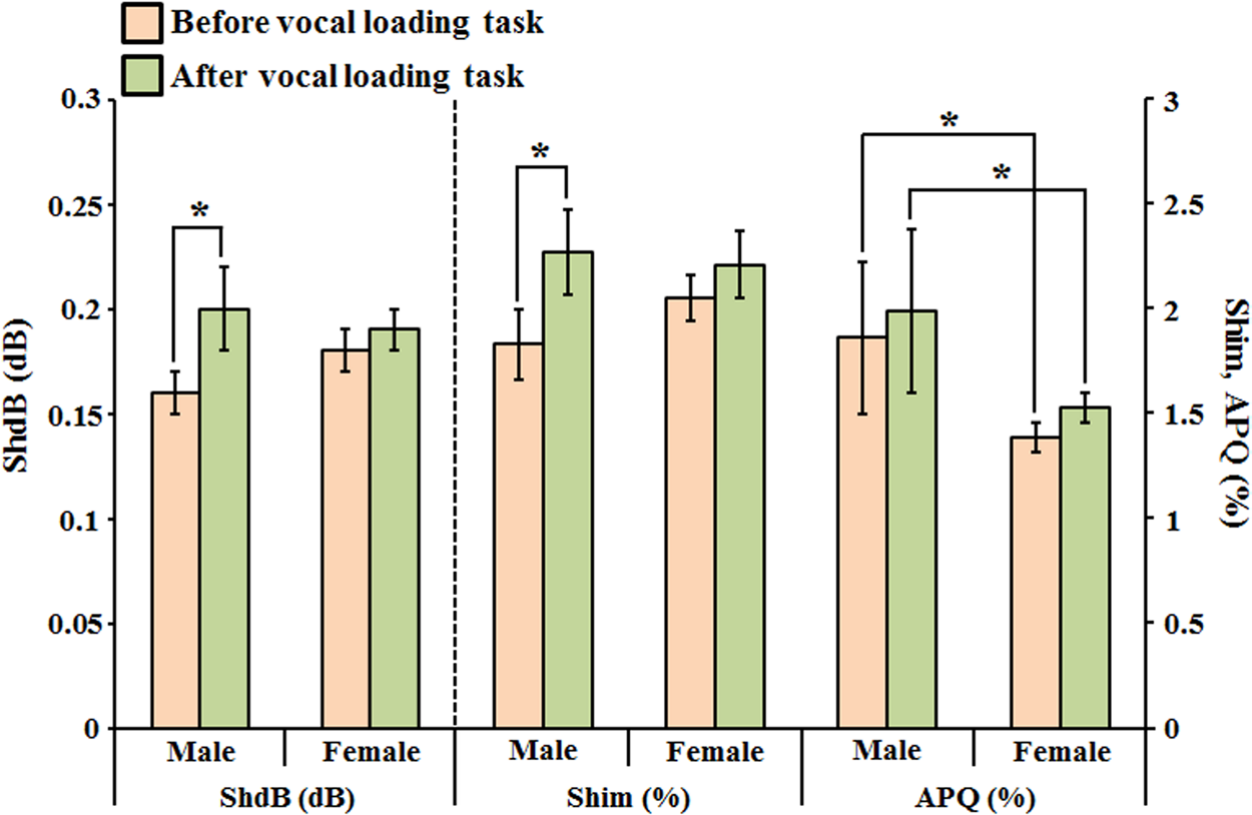
The analysis of the variation of parameters related to amplitude perturbation before and after vocal loading task in male and female groups. (two sample *t*-test *p < 0.01).

Likewise, the acoustic parameters related to amplitude perturbation were also calculated to evaluate the differences between male and female groups after and before vocal loading task. Figure 5 shows the variation of the parameters related to amplitude perturbation. The result indicated that all the parameters were not significantly different between male and female groups except the parameter of APQ. Besides, there were only ShdB and Shim presented significantly different before and after vocal loading task in the male group. It is also noteworthy that all the value of parameters of amplitude perturbation increased after vocal loading task.

**Figure 6:**
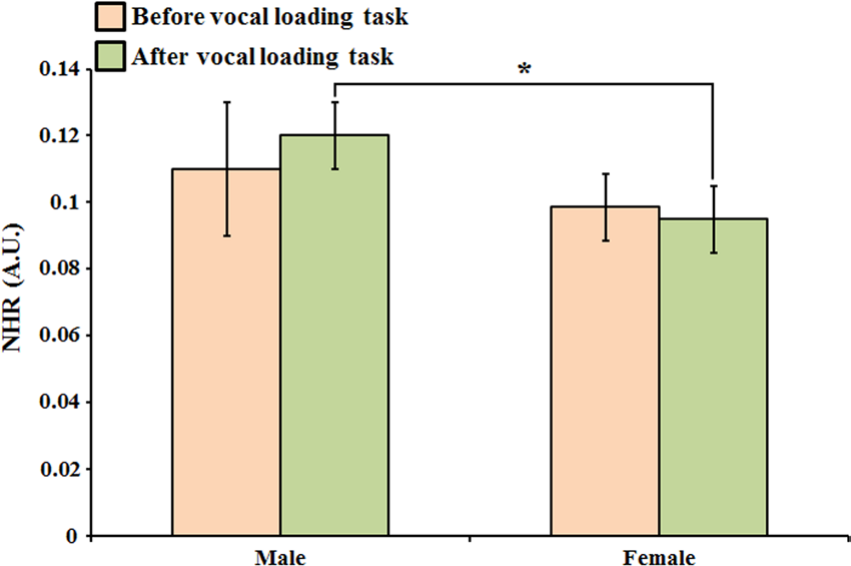
The analysis of NHR variation before and after vocal loading task in male and female groups. (two sample t-test, *p < 0.01).

Figure 6 shows the variation of noise-to-harmonic ratio (NHR) before and after vocal loading task in the male and female groups. The result presented significantly different between the male and female groups after vocal loading task. The value of NHR was not significantly different before and after vocal loading task in the male group or in the female group. Besides, the value of NHR increased after vocal loading task in the male group, and slightly decreased after vocal loading task in the female group.

## Discussion

Acoustic analysis is the non-invasive and most used approach to voice quality assessment of voice disorder in clinical research and application^36–40^. Unlike previous studies of acoustic analysis that used the traditional microphone to record voice sample, the present study developed and used an optical technique (as an optical microphone) to record voice sample for acoustic analysis. This method was used to receive the vibration signals transmitted from the vocal cords to the surface of the tissue. The greatest benefit of the optical microphone is to avoid ambient sound and electromagnetic interference.

The spectrum analysis of signals in Fig. 2 showed that the optical method can indeed reconstruct the voice signal correctly. Additionally, the results of acoustic analysis of optical signal were also used to prove the feasibility of this method. In the Fig. 3, the result of average fundamental frequency in the male group was lower than in the female group that showed significant gender differences. Our result corresponds with several previous studies that also revealed lower fundamental frequency in healthy male group and significant gender differences^41–46^. The results implied that the pitch is affected by the size of vocal cords. The size (length) of vocal cords is larger in the adult male group than in the female group^47,48^. This is the reason why adult male voices are usually deeper and present lower fundamental frequency than female voices. In fact, the decreased fundamental frequency might not really reveal the poor voice quality but it is really correlated with the swelling of vocal cords and decreased mucosa wave because of the swelling of Ranke’s space. There might be more swelling even some transient inflammatory response to vocal cords and cause decreased mucosa wave. Chronic vocal trauma even causes fluid accumulation in Ranke’s space or higher collagen formation and decreased thus decreased vocal vibration and cause decreased fundamental frequency. Our results also showed that the fundamental frequency was significantly lower after vocal loading task in male group. Although the fundamental frequency was not significantly lower after vocal loading task in the female group, the fundamental frequency still decreased after vocal loading task. This finding implies that the voice quality changes (the voice sound “hoarser” and “deeper” after vocal loading task) in the male group more than in the female group after the same strength of the vocal loading task.

The increased frequency perturbation is responsible for hoarse, harsh or rough voice quality. Our results showed that all the parameters of frequency perturbation presented significant gender differences (as shown in Fig. 4). The parameters of frequency perturbation were higher in the male group than in the female group. These results implied that the adult male voices are rougher than female voices. Although the parameters of frequency perturbation were not significantly different after vocal loading task in all groups, the parameters still increased after vocal loading task. The results implied the worse vocal stability after vocal loading task. Same as frequency perturbation, the amplitude perturbation was used to evaluate the vocal stability. In the Fig. 5, the parameters of amplitude perturbation were not significantly different between male and female groups except APQ. The results are consistent with the previous study that used traditional voice assessment devices^42^. The ShdB and Shim were significantly higher after vocal loading task in the male group. It is noteworthy that all the parameters increased after vocal loading task. The results also implied the worse vocal stability after vocal loading task. The result of the NHR presented a significant difference between male and female groups after vocal loading task. This finding also implies that the voice quality decreases in the male group more than in the female group after the vocal loading task.

The acoustic analysis may be influenced by several factors include age, gender, vowel phonation, and vocal intensity^41–46^. Besides, acoustic analysis cannot be simply compared in different speech analysis software^42^. As mentioned before, voice disorder is a complex multifaceted clinical problem. The multidimensional assessment is very important for clinical applications of voice disorder. Additionally, oxidative metabolism may play a very important factor in vocal fatigue. Therefore, the oxidative metabolism was simultaneously detected before and after vocal loading task in this study. According to the result of previous study, hypoxia of vocal cords was induced by vocal overuse^49^. Our results demonstrated the vocal cord hypoxia after vocal loading task both in male and female groups (as shown in Fig. 1). It is noteworthy that the oxygen recovery was faster in the female group. This result was consistent with our results of the optical acoustic analysis that suggested the voice quality decreases in the male group more than in the female group after vocal loading task. Besides, this finding also implied that the recovery rate of oxygen consumption may relate to the size/structure of vocal cords. Therefore, integration of oxidative metabolism detection and acoustic assessment by using optical technique may provide more information for assisted diagnosis of vocal disorders.

## Methods

### Participants

In this study, 60 healthy adults (30 males and 30 females), age between 20 to 25 years old (the mean age was 22 ± 2.15 years old) without throat or vocal cord diseases at least one month, were recruited from National Chiao Tung University, Taiwan. All participants provided written informed consent. The study was in accordance with the latest version of the Declaration of Helsinki, and approved by the Institutional Review Board (IRB) in Research Ethics Committee for Human Subject Protection, National Chiao Tung University, Taiwan.

### Experimental Setup

**Figure 7:**
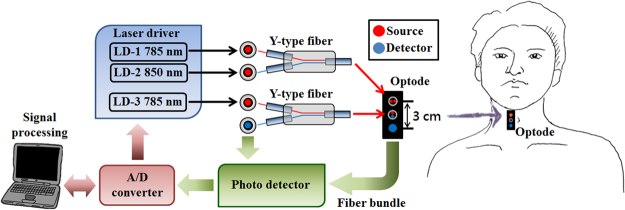
Multi-functional vocal cords detection system. LD-1 and LD-2 were used to calculate the concentration changes in oxygenated hemoglobin (Δ[HbO_2_]) and deoxygenated hemoglobin (Δ[Hb]) for oxygen consumption evaluation; LD-3 was used to reconstruct the optical acoustic signals for acoustic analysis.

In this study, the non-invasive oxidative metabolism detection and acoustic assessment were integrated into a novel system by using the fNIRS-DOT, that we called it a multi-functional vocal cords detection system. Therefore, the information of oxidative metabolism and acoustics of the vocal cords could be captured simultaneously. Figure 7 shows the scheme of multi-functional vocal cords detection system. The system setup included a pair of laser diodes (785 nm and 850 nm) (WSLR-785/850–050 m-M-PD, Wavespectrum, Beijing, China), an independent laser diode (785 nm) and two silicon photo detectors (PDA100A, Thorlabs, Newton, New Jersey, U.S.). In function of the oxidative metabolism detection, the bifurcated fiber bundles, also called Y-type fibers (BFY400HS02, Thorlabs, New Jersey, US) was used to introduce laser light from the two laser heads into one optical fiber as the same emission point. The source-detector separation is 3 cm that can provide adequate depth of penetration (the penetration depth into tissue is approximately 1.5 cm or larger^26–29^) for oxidative metabolism detection of the vocal cords. In function of the acoustic assessment, the Y-type fiber was used to transmit and receive the optical signal as an optical microphone to detect the signal of the tissue surface vibration. All the backscattered optical signals from vocal cords and tissue surface vibration were detected with two silicon photo detectors (Si-PD), respectively. The amplified analog signals from the Si-PD were converted to digital data by using an A/D converter (NI myRIO-1900, National Instruments, Austin, Texas, U.S.) with 12-bit resolution. The A/D converter was also used to regulate the optimum laser diode drive current with the technique of Time-Division Multiplexing (TDM) to obtain stable and accurate measurements. The optical power of laser diode was regulated at 5 mW. The optical fibers were fixed on a black flexible probe that was brought in close contact with the skin to prevent noise from the environmental and surface backscattering.

### Experimental Protocol and Data Acquisition

**Figure 8:**
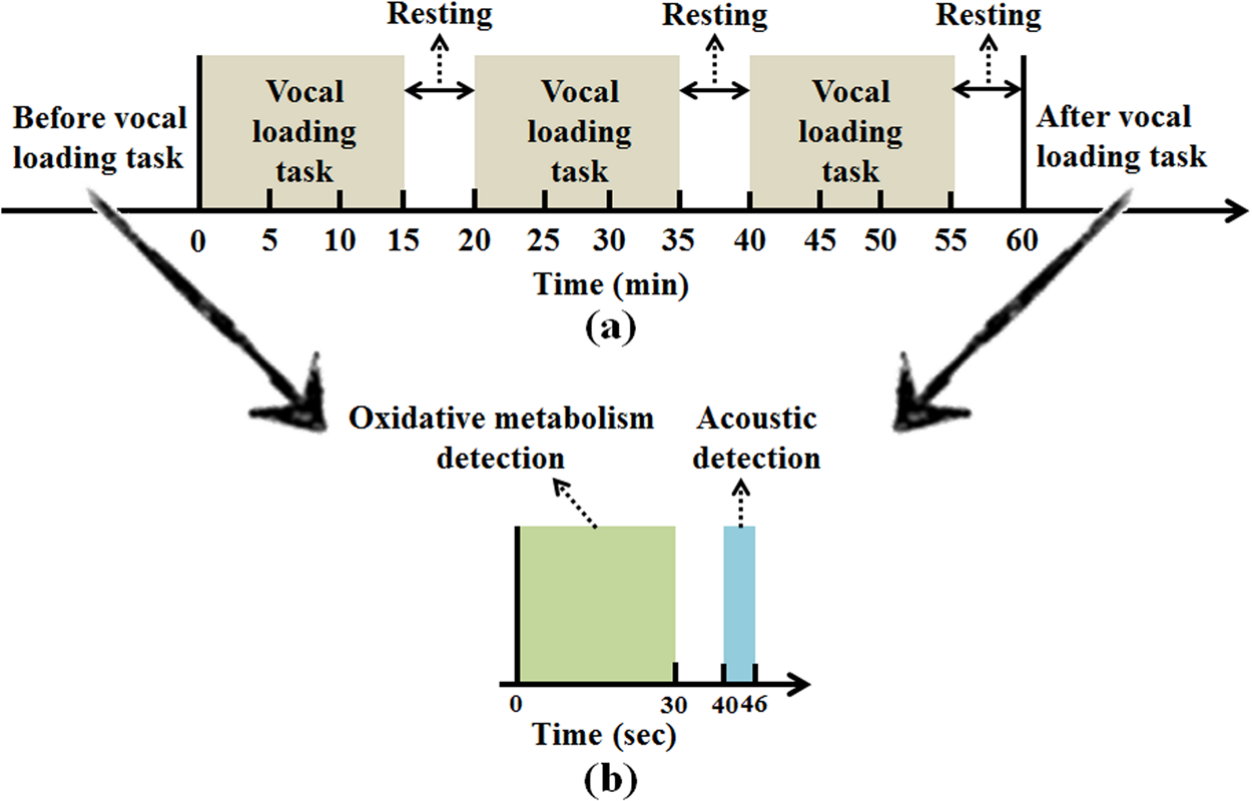
The protocols of vocal loading task and data acquisition. (**a**) the protocol of vocal loading task; (**b**) the protocol of the oxidative metabolism detection and optical acoustic data acquisition.

Figure 8 shows the protocols of vocal loading task and optical signals measurement. During the experiment, subjects sat on a comfortable chair in a silent room. A totally 60-min vocal loading task consists of three 15-min intervals and 5-min vocal resting state between each task (as shown in Fig. 8(a)). During the vocal loading task, subjects were asked to read aloud at a level approximately 90 dB and a digital sound level meter was used to verify. According to previous study, this protocol could trigger a mild inflammation of the vocal cords^13,14^. Therefore, the optical measurements were performed before and after vocal loading task according to the following three steps: 1) oxidative metabolism detection for 30-sec; 2) acoustic detection after a 10-sec separation for 6-sec (as shown in Fig. 8(b)). In the step of oxidative metabolism detection, subjects kept in the vocal resting state for measurement. In the step of acoustic detection, subjects were asked to voice the sustained vowel /a/ at a flat tone and comfortable pitch that without breaks for at least 6 seconds.

## Data Analysis

### Oxidative metabolism detection

In this function, the backscattered optical signals of the dual-wavelength (785 nm and 850 nm) from human tissue were received for calculating the concentration changes in Δ[HbO_2_] and Δ[Hb] with technique of continuous-wave and a sampling rate of 50 Hz. According to the modified Beer-Lambert Law (MBLL)^25,50,51^, the optical density (OD) can be defined as follows:

1 $$O{D}_{\lambda }=-\,{\mathrm{log}}_{10}(\frac{I}{{I}_{0}})={\varepsilon }_{\lambda }CL{B}_{\lambda }+{G}_{\lambda }$$

where *I*_*o*_ and *I* are the intensities of incident light and detected light, respectively; The *OD*_*λ*_ is the optical density for wavelength *λ* that means the attenuation of near-infrared light intensity in tissue; *ε*_*λ*_ is the extinction coefficient of the chromophore; *C* is the concentration of the chromophore; *L* is the distance between the light entry and exit the tissue; *B*_*λ*_ is a pathlength factor that related to tissue scattering; *G*_*λ*_ is defined as a geometric factor that related to tissue geometry. Assuming that *G*_*λ*_ remains constant during a measurement, the change in optical density Δ*OD*_*λ*_ can be obtained as follows:

2 $${\rm{\Delta }}O{D}_{\lambda }=O{D}_{\lambda }(t)-O{D}_{\lambda }({t}_{0})=-\,{\mathrm{log}}_{10}(\frac{I(t)}{I({t}_{0})})={\varepsilon }_{\lambda }{\rm{\Delta }}CL{B}_{\lambda }$$

where *OD*_*λ*_(*t*_0_) and *OD*_*λ*_(*t*) are the initial and instantaneous values of the optical density from the tissue, respectively; *I*(*t*_0_) and *I*(*t*) are the measured intensities at initial and instantaneous time; Δ*C* is the change in concentration of the chromophore. In human tissue, changes in concentration were dominated with the chromophore of HbO_2_ and Hb. Therefore, the change in optical density can be obtained as follows:

3 $${\rm{\Delta }}O{D}_{\lambda }=({\varepsilon }_{\lambda }^{Hb{O}_{2}}\cdot {\rm{\Delta }}[Hb{O}_{2}]+{\varepsilon }_{\lambda }^{Hb}\cdot {\rm{\Delta }}[Hb])\cdot L{B}_{\lambda }$$

The concentration changes in Δ[HbO_2_] and Δ[Hb] could be obtained by solving Eq. (3) with the optical signals of dual-wavelength (785 nm and 850 nm). Assuming that *L* and *B*_*λ*_ remains constant during a measurement, the change in optical density can be obtained as follows:

4 $${\rm{\Delta }}O{D}_{785}=({\varepsilon }_{785}^{Hb{O}_{2}}\cdot {\rm{\Delta }}[Hb{O}_{2}]+{\varepsilon }_{785}^{Hb}\cdot {\rm{\Delta }}[Hb])$$

5 $${\rm{\Delta }}O{D}_{850}=({\varepsilon }_{850}^{Hb{O}_{2}}\cdot {\rm{\Delta }}[Hb{O}_{2}]+{\varepsilon }_{850}^{Hb}\cdot {\rm{\Delta }}[Hb])$$

Finally, the description can be rewritten from Eq. (4) and Eq. (5) as follows:

6 $${\rm{\Delta }}[Hb{O}_{2}]=\frac{{\varepsilon }_{785}^{Hb}\cdot {\rm{\Delta }}O{D}_{850}-{\varepsilon }_{850}^{Hb}\cdot {\rm{\Delta }}O{D}_{785}}{{\varepsilon }_{785}^{Hb}\cdot {\varepsilon }_{850}^{Hb{O}_{2}}-{\varepsilon }_{850}^{Hb}\cdot {\varepsilon }_{785}^{Hb{O}_{2}}}$$

7 $${\rm{\Delta }}[Hb]=\frac{{\varepsilon }_{850}^{Hb{O}_{2}}\cdot {\rm{\Delta }}O{D}_{850}-{\varepsilon }_{785}^{Hb{O}_{2}}\cdot {\rm{\Delta }}O{D}_{785}}{{\varepsilon }_{785}^{Hb}\cdot {\varepsilon }_{850}^{Hb{O}_{2}}-{\varepsilon }_{850}^{Hb}\cdot {\varepsilon }_{785}^{Hb{O}_{2}}}$$

In this study, the concentration changes of Δ[HbO_2_] and Δ[Hb] were used to analyze the oxidative metabolism of vocal cords before and after vocal loading task.

### Acoustic assessment with optical microphone

In this function, the backscattered optical signal of the independent laser diode (785 nm) from the tissue surface vibration was detected with a sampling rate of 50 kHz during subjects voiced the sustained vowel /a/. The sustained vowels /a/, /u/, /o/, and /i/ are usually used in clinical acoustic assessments. However, the sustained vowel /a/ would enhance measurement reliability^43^. The spectrum distribution was calculated with the algorithm of fast Fourier transform (FFT). Additionally, the software of Multi-Dimensional Voice Program (MDVP) was utilized to analyze voice parameters included average fundamental frequency, frequency perturbation (Jita, Jitt, PPQ, and RAP), amplitude perturbation (ShdB, Shim, and APQ), and noise-to-harmonic ratio (NHR)^52–55^. The choice of these voice parameters were designated by the clinician. The description of each of the extracted parameter was listed as follows: Average fundamental frequency (f_0_, Hz): f_0_ is an average value of all extracted period-to-period fundamental frequency value that is calculated from the extracted period-to-period pitch data; Absolute jitter (Jita, µsec): To calculate the period-to-period variability of the pitch period that dependent on the average fundamental frequency. Therefore, Jita is typically related to hoarse voices; Jitter percent (Jitt, %): Jitter percent is a relative measure of a very short-term variability of the pitch period. It also related to hoarse voices. The higher value of Jita and Jitt means unstable of voice quality; Pitch perturbation quotient (PPQ, %): PPQ is a relative measure of pitch perturbation in short term cycle (smoothing cycle of 5 periods) of voice analysis. Hoarse and breathy voices may cause PPQ to increase; Relative average perturbation (RAP, %): RAP is a relative measure of average pitch perturbation in a short-term cycle (smoothing cycle of 3 periods) of voice analysis. Hoarse and breathy voices also may cause PPQ to increase; Shimmer in dB (ShdB, dB): To calculate in dB of the very short term variability of the peak-to-peak amplitude of the voice that is very sensitive to amplitude variations between consecutive pitch periods. It is also related to hoarse and breathy voices; Shimmer percent (Shim, %): Shim is a relative measure of a very short-term variability of the peak-to-peak amplitude of the voice. It is typically related to hoarse and breathy voices; Amplitude perturbation quotient (APQ, %): APQ is a relative measure of a short term (smoothing cycle-to-cycle of 11 periods) irregularity of the peak-to-peak amplitude of the voice. Hoarse and breathy voices may also cause APQ to increase; Noise-to-harmonics ratio (NHR, A.U.): NHR is defined as the ratio of the energy of noise and the energy of harmonic spectral during the range of 70–4200 Hz. The lower value of NHR indicates the better voice quality. Contrarily, the higher value of NHR is interpreted as more spectral noise that may be caused by the frequency and amplitude variations, turbulent noise, subharmonic components or voice breaks.

In the both two functions, the average data was obtained for group-level analysis to reduce the effects of individual differences. The results were expressed as the mean ± SD. The significant differences analysis between healthy male and female group was made with a two-sample *t*-test. Additionally, the significant difference analysis before and after vocal loading task was also made with a two-sample *t*-test. Analyses were performed with software of LabVIEW (Version 2017, National Instruments, Austin, Texas, U.S.), MATLAB (Version 7.11.0.584 R2010b, MathWorks Inc., Natick, MA, U. S.) and Multi-Dimensional Voice Program (Version MDVP model 5150, Lincoln Park, NJ, U.S.). A more stringent *p* value of <0.01 was considered as statistically significant in two-sample *t*-test.

## Conclusion

In this study, the multi-functional vocal cords detection system was developed by using optical technique. This system can provide a novel and non-invasive detection approach for human vocal fold oxidative metabolism detection and acoustic assessment simultaneously. Our results demonstrated that the physiological analysis of vocal cords (include oxidative metabolism detection and acoustic assessment) before and after vocal loading task could be successfully measured by using an optical method. Therefore, this optical method could be a potential tool for clinical application of voice disorder. Although there are still several limitations^31^, fNIRS could provide relevant information on key mechanisms of oxidative metabolism of different organs by performing a clear and reliable hypothesis of the test protocol. In the future study, the system will be optimized for more clinical applications such as postoperative prognosis and monitoring. We hope that the proposed method can provide more clinical information to help ENT physician to develop the treatment strategy and therapeutic monitoring of vocal disorders.
